# Mitophagy plays a central role in mitochondrial ageing

**DOI:** 10.1007/s00335-016-9651-x

**Published:** 2016-06-28

**Authors:** Alan Diot, Karl Morten, Joanna Poulton

**Affiliations:** Nuffield Department of Obstetrics and Gynaecology, The Women’s centre, University of Oxford, Oxford, OX3 9DU UK

## Abstract

The mechanisms underlying ageing have been discussed for decades, and advances in molecular and cell biology of the last three decades have accelerated research in this area. Over this period, it has become clear that mitochondrial function, which plays a major role in many cellular pathways from ATP production to nuclear gene expression and epigenetics alterations, declines with age. The emerging concepts suggest novel mechanisms, involving mtDNA quality, mitochondrial dynamics or mitochondrial quality control. In this review, we discuss the impact of mitochondria in the ageing process, the role of mitochondria in reactive oxygen species production, in nuclear gene expression, the accumulation of mtDNA damage and the importance of mitochondrial dynamics and recycling. Declining mitophagy (mitochondrial quality control) may be an important component of human ageing.

## Introduction

Ageing of multi-cellular organisms is a highly complex biological process associated with a progressive decline in the performance of most organs, culminating in the inability to meet environmental demands for survival (Linnane et al. 1989). At a cellular level, it is the process during which cells accumulate damage and changes that affect their function. These include changes in: oxidative stress, epigenetic status, energy availability, telomeres, loss of stemness, mitochondrial damage and impaired cell cycle (Wei et al. 1998; Lardenoije et al. 2015; Fyhrquist et al. 2013; Goodell and Rando 2015). These insults amount to cellular senescence, which halts the proliferation of damaged or dysfunctional cells and constrains the malignant progression of tumour cells (Takeuchi et al. 2010).

Lopez-Otin used three simple criteria to define hallmarks of ageing: (1) manifestation during normal ageing; (2) experimental aggravation accelerating ageing; and (3) experimental amelioration slowing down ageing. According to this study, the nine hallmarks of ageing fulfilling these criteria are genomic instability, telomere attrition, loss of proteostasis, deregulated nutrient sensing, altered intercellular communication, cellular senescence, stem cell exhaustion, epigenetic alterations and mitochondrial dysfunction (Lopez-Otin et al. 2013).

In tissue culture, senescent cells become larger following a permanent arrest in cell cycle, due to p53 and pRB pathways after an attempt to repair cell damage (Campisi 2005). If such non-dividing cells accumulate in vivo, they will presumably impair the renewal and repair capacity of the tissue. Moreover, senescence upregulates the secretion of factors such as Interleukins (IL-6, IL-7), chemokins (IL-8), growth factors (VEGF, HGF, SCF), receptors and ligands (ICAM-1 and 3, EGF-R, Fas) that may vary between tissues but affect their structure and function (Takeuchi et al. 2010; Coppe et al. 2010). This causes deterioration in the physiological functions of the organism, a decline in physical performance due to the decrease in aerobic capacity and in the strength of skeletal muscles, (Hebert et al. 2015) and eventually death. It is associated with frailty, cardiovascular, neurodegenerative and other age-related disease. The rising number of senescent cells is accompanied by an increased sensitivity to apoptosis (Tower 2015) which may explain the development of these degenerative disorders. Targeting cells expressing the senescence biomarker p16^Ink4a^, which activates pRB to stop cell growth, limits the accumulation of senescent cells, hence delaying the appearance of age-related pathologies and improving exercise tolerance (Baker et al. 2011). The increased sensitivity of senescent cardiomyocytes to apoptosis leads to cardiac pathologies such as hypertrophy, fibrosis, and diminished contractility. Age-related neurodegenerative disease such as Alzheimer, Parkinson and amyotrophic lateral sclerosis are all associated with accumulating aggregates of misfolded protein (reviewed in Skovronsky et al. 2006). These can not only activate the unfolded protein response (UPR) but also lead to calcium dysregulation and to less energy availability due to mitochondria dysfunction. They may increase apoptosis, cause neuronal loss and damage synapses.

Mitochondrial function plays a major role in many cellular pathways such as ATP production, calcium regulation, apoptosis and nucleotide synthesis. Recently, mitochondria have been implicated in nuclear gene expression and epigenetic alterations (Guantes et al. 2015; Muir et al. 2016). They are thus even more likely to play a central role in ageing than was previously believed. For instance, work in *S. cerevisiae* links optimal mitochondrial quality with longer lifespan (Higuchi et al. 2013). Mitochondria have been shown to be involved in telomere length, maintenance of pluripotency, genomic instability, cellular senescence and epigenetic alterations (Ahlqvist et al. 2015; Correia-Melo and Passos 2015; Minocherhomji et al. 2012; Monickaraj et al. 2012; Tyrka et al. 2015). Mitochondrial DNA (mtDNA) damage has been widely discussed in this context (Linnane et al. 1989). Recent advances in understanding of mitochondrial quality control add an important dimension to the study of ageing. Mitophagy is one of several processes that maintain mitochondrial quality, but is the only one known to turn over whole mitochondrial genomes. While relatively unimportant in bulk turnover of all mitochondrial components (Kim et al. 2012), it may play a critical role in the accumulation of mtDNA damage and potentially ageing (Payne et al. 2013; Twig et al. 2008). Furthermore, autophagy may be an important determinant of stem cell pluripotency (Phadwal et al. 2012) and be attenuated in several neurodegenerative diseases (Nixon 2013).

Because of its importance, this review will therefore focus on the mitochondrial aspects of ageing. We will touch on a range of organisms but focus on mammalian systems and human ageing in particular. We will discuss how mitochondria can have such an impact, focussing on the metabolic side, the role of mitochondria in reactive oxygen species (ROS) production, in nuclear gene expression and heteroplasmic mtDNA damage (where damaged and intact mtDNA co-exist), and finally how mitochondrial dynamics and recycling can affect the ageing process.

## MtDNA heteroplasmy and ageing: the vicious circle hypothesis

The causes of ageing have been discussed for decades, and the advances in molecular and cell biology have provided important insights into this area. It has become clear that mitochondrial function declines with age, and the emerging concepts suggested novel mechanisms. MtDNA was identified, characterised and shown to play a role in human disease. Thousands of copies of mtDNA are present in most types of cells. In normal individuals, these are identical, but in heteroplasmic mtDNA disease, normal and mutant mtDNA co-exist in the same cells. In these diseases, the dose of mutant mtDNA may increase with time, causing progressively worsening symptoms. Low levels of heteroplasmic mtDNA mutants may be present in symptom-free maternal relatives. These findings soon suggested an mtDNA-based theory of ageing in which an increase in mitochondrial heteroplasmy may underlie the decline in energy in ageing individuals. Furthermore, the uniparental inheritance of mtDNA results in a selection asymmetry that may explain the shorter lifespan of males than females: mtDNA mutations that affect only males will not respond to natural selection, imposing a male-specific disadvantage that results from mitochondrial mutation load (Wolff and Gemmell 2013).

However, because there is no clear maternally inherited determinant of life-expectancy, somatic accumulation of mtDNA damage in males could contribute to their early death (Linnane et al. 1989). For instance, mitochondria in human fibroblasts harbour mtDNA mutations at levels that increase with the age of the donor, and these confer a respiratory chain defect (Laderman et al. 1996). Furthermore, mtDNA mutations can be found in multiple different tissue types at levels that increase with age (Simonetti et al. 1992; Bender et al. 2006; Cortopassi and Arnheim 1990; Melov et al. 1999; Marin-Garcia et al. 2006) and may be associated with different types of neurodegeneration such as Alzheimer (Coskun et al. 2004) and Parkinson’s disease (Bender et al. 2006). MtDNA deletions that are characteristic of diseases due to defects in mtDNA maintenance and accumulate in post-mitotic tissues of ageing humans are found at increased levels in many neurodegenerative diseases (Krishnan et al. 2008). Furthermore, these recapitulate the accumulation of mtDNA rearrangements that appear to cause senescence in fungi (Osiewacz and Hermanns 1992). Several studies focussing on the control region of human mtDNA showed that mutations accumulate in this region in muscle from 30-year olds, but not in brain (Calloway et al. 2000; Murdock et al. 2000). These are de novo mutations as they are detected in grandmothers but not in grandchildren of the same family (do Rosario Marinho et al. 2011). It is to be noted that having a mitochondrial condition does not seem to worsen this effect (da Costa et al. 2007). An array-generated single-nucleotide polymorphism study has shown that even if overall the heteroplasmy increases with age, some sites lose variation while some increase it (Sondheimer et al. 2011). In the hypervariable segment 1, especially at nucleotides 16189, 16304, and 16311, heteroplasmy seems to increase with age (Pliss et al. 2011). This may well reflect any effect that these polymorphisms have on mitochondrial function: investigation of mtDNA heteroplasmy in platelets from 137 people revealed an association between increasing low level heteroplasmy with age for known pathogenic mutations and ageing phenotypes (Tranah et al. 2015). In a mouse model of atherosclerosis, it has been shown that mtDNA deletions associated with low COX III protein levels appear long before atherosclerosis plaques (Tian et al. 2016). This study shed also some light on the reason for a decrease in mtDNA quality since it shows that OGG1, a mitochondrial enzyme involved in mtDNA excision, decreases with age.

If the accumulation of mtDNA mutations led to a compensatory increase in mtDNA copy number, the deletion mutants would accumulate (Elson et al. 2001). Related ideas have been discussed widely (Linnane et al. 1989; Melov et al. 1999; Corral-Debrinski et al. 1992; Cortopassi et al. 1992; Osiewacz and Hermanns Osiewacz and Hermanns 1992; Blanchard et al. 1993; Munscher et al. 1993; Takeda et al. 1993; Tritschler and Medori 1993; Birch-Machin et al. 1998) and led to the well-known vicious cycle theory of ageing. This proposes that mitochondrial dysfunction or oxidative stress caused by accumulation of mtDNA mutants in itself damages mitochondria further. In its simplest form, this has little supporting data (Tengan et al. 1997), but it gathered credibility because of the surprising phenotype of a mouse which was engineered to study the effect of rapid accumulation of mtDNA mutations as a result of impaired proof reading in the mitochondrial gamma polymerase. This Polg “mutator” mouse manifests a premature ageing phenotype (Trifunovic et al. 2004). As mentioned earlier ROS levels increase with age, especially mitochondrial ROS, and this increase in oxidative stress could be responsible for de novo mtDNA mutations that might feed the ageing process (Mikhed et al. 2015). Thus, oxidative stress/ROS produced by the mitochondrial electron transport chain is held to damage the mitochondria leading exponentially to more ROS production and mitochondrial damage. However, the predicted exponential increase in mutation load is not apparent (Trifunovic and Larsson 2008). Hence, a more sophisticated version of the vicious circle hypothesis is needed, and one that takes spatial relationships into account provides more answers (Kirkwood and Kowald 2012). Because mitochondrial nucleoids are in close proximity to inner mitochondrial membrane and the respiratory chain, the main source of cellular ROS, they are highly susceptible to ROS-induced damage. Hence, external oxidative stress would likely have an insignificant effect on mtDNA damage, even when it substantially increases protein oxidation. This would also explain why simply manipulating general anti-oxidant concentrations does not robustly rescue the damage (Kirkwood and Kowald 2012). It is further supported by a tissue culture study showing that the age-associated decrease in cell spreading induces an increase in ROS production and an increase in mtDNA mutations (Quan et al. 2015). Moreover telomere length may be linked to the mtDNA content and quality (Monickaraj et al. 2012; Tyrka et al. 2015). How they relate to each other remains to be determined. In one scenario, telomerase protects mitochondria from mild oxidative stress (Ahmed et al. 2008). Other authors suggest that p53 repression of PGC-1 promoter due to a telomere dysfunction could be involved or that TERT affects mtDNA repair (Monickaraj et al. 2012; Tyrka et al. 2015).

Many studies have investigated transmission of heteroplasmy, distinguishing between selection in the germline and in somatic tissues with rapid turnover, such as intestinal crypts. Low-level heteroplasmy and non-pathogenic mutations are readily transmitted in the germline (Sondheimer et al. 2011; Giuliani et al. 2014; Greaves et al. 2014). Pathogenic mutations tend arise in somatic cells rather than the germline, only becoming apparent in early adulthood. This is entirely consistent with purifying selection during mtDNA transmission (Greaves et al. 2014). Interestingly, next-generation sequencing showed that the mtDNA mutation rate does not seem to increase with age (Greaves et al. 2014). This suggests that ageing affects other processes, such as mtDNA turnover, and that these underline the accumulation of mutant mtDNA post-mitotic tissues. Perhaps the best evidence to support the mitochondrial theory of ageing is the finding that passive transmission of mtDNA mutations, generated by Polg mutations in preceding generations, can induce a mild ageing phenotype in the absence of an ongoing defect in mtDNA maintenance (Ross et al. 2013).

Our recent study shows that mitophagy in skin fibroblasts declines as the age of the donor increases. Moreover, forcing cells to rely on their mitochondria to produce energy induces mitophagy that specifically eliminates mutant mtDNA (Diot et al. 2015). This may contribute to the improvement of mitochondrial function and the beneficial effect of exercise (Romanello and Sandri 2015) and caloric restriction in delaying the ageing process and concomitant deterioration in mitochondrial function. The increase of sirtuin 3 localised to mitochondria and involved in mitochondrial ROS detoxification, ATP production and network dynamics (Jacobs et al. 2008; Ahn et al. 2008; Samant et al. 2014; Hirschey et al. 2010; Jing et al. 2013; Papa and Germain 2014), during exercise or the activation of mitophagy following caloric restriction would trigger the clearing of mutant mtDNA. This should reduce the level of heteroplasmic mutant mtDNA and improve mitochondrial quality and function.

## Mitochondrial function, ROS production and ageing

Declining mitochondrial activity is a well-known feature of ageing (Hebert et al. 2015). Recent studies have shown that mitochondrial dysfunction has a role to play in senescence and in the ageing phenotype of the immune system. In dendritic cells, mitochondrial membrane potential, the ATP turnover and mitochondrial respiratory chain (MRC) coupling and OXPHOS decrease with age (Chougnet et al. 2015). These results were mimicked pharmacologically in young dendritic cells. Other studies have shown that, with ageing, oxygen consumption and activities of complex III and complex IV are decreased (Das and Muniyappa 2013; Ben-Meir et al. 2015), and that this can be rescued with coenzyme Q supplementation (Ben-Meir et al. 2015; Varela-Lopez et al. 2015). This has been observed in several tissues in human and mice model such as granulosa cells (Ben-Meir et al. 2015), muscle (Porter et al. 2015) and alveolar bone (Varela-Lopez et al. 2015). Impaired mitochondrial function may well be important in age-related insulin resistance (Petersen et al. 2015; Goldsworthy and Potter 2014) and in the tendency for failing hearts to use glycolytic rather than oxidative substrates (Evans and Clarke 2012). Further, dysfunctional mitochondria may be a source of oxidative stress.

The free radical theory of ageing was first established in the 1950s by Denham Harman (Harman 1956). While excessive ROS cause oxidative stress, lower levels are critically important in cell signalling (Schieber and Chandel 2014). ROS production increases with age and has been linked to mtDNA mutations accumulation, protein oxidation, shorter telomere, and increased apoptosis (Mikhed et al. 2015; Carney et al. 1994; Salpea et al. 2013; Wang et al. 2013). As a proof of concept, work in yeast has shown that feeding yeast with lithocholic acid, a compound acting on redox processes, delays ageing. It results in an enlargement of mitochondria and an increase in the efficiency of mitochondrial respiration (Burstein and Titorenko 2014). Similarly, reducing ROS by targeting electron scavengers to mitochondria improves the phenotype of sarcopenia in rats, increasing contractility, reducing protein oxidation and increasing activity of CI, CIII, and CIV (Javadov et al. 2015). However, it is the level of oxidative stress rather than of ROS production that is important. Genetic knockdown of NRMT1, a protein involved in protein–DNA interaction, increases ROS and thus shortens lifespan in the mouse (Bonsignore et al. 2015). When livers from survivors were analysed, they revealed a decreased ROS production demonstrating that it is the decreased capacity to deal with ROS damage that causes problems in this mouse. Again, genetic reduction in SOD1 activity also exacerbates ageing in mice, and these display features of altered neurotransmitter release and decreased calcium influx (Ivannikov and Van Remmen 2015). The Polg mutator mouse model also displays an impaired anti-oxidant defence system, with reduced ergothioneine levels or carnitine, which could result from a depletion in mitochondria (Clark-Matott et al. 2015). Neurotransmission in this mouse is also altered, indicating a potential link between ROS and neurodegeneration. Similarly, increasing mitochondrial oxidative stress in stem cells leads to senescence, cell cycle arrest and loss of stemness (Velarde et al. 2015). When these mice reach old age, this results in a thinner epidermis and delays wound closure (Velarde et al. 2015), characteristics of an ageing phenotype. In humans, this oxidative mechanism could be tissue specific as a study in related women has shown that in muscle mitochondrial function decreases with ageing, together with a drop in MRC complex proteins and mtDNA copy number (Hebert et al. 2015). This age-related decline in mitochondrial function correlates with a decreased expression with age of Sirtuin 3 (see later section) and both can be rescued by exercise (Lanza et al. 2008).

At the cellular level, hematopoietic stem cells from aged mice have an increased ROS levels associated with DNA damage, apoptosis and shorter telomeres. This results in a decreased capacity for regeneration (Porto et al. 2015). This increase in ROS level with age, which also has a circadian rhythm (Gong et al. 2015), could be due to a dysregulation of mitochondrial gene expression.

However, this ROS ageing theory has been increasingly challenged and now requires revision.

Indeed, some studies showed that although there is a correlation between mtROS production and lifespan, the mtROS are not directly responsible for the ageing. They showed, however, that the mtDNA controls longevity (Sanz et al. 2010). Similarly a knockdown of CCO-1, the nuclear-encoded cytochrome c oxidase-1 subunit Vb/COX4, induced a moderate alteration in the electron transport chain, ultimately leading to an increased longevity (Durieux et al. 2011; Yang and Hekimi 2010). These experiments showed that although the generation of superoxide is increased, the overall ROS level is not. Moreover, the increase in superoxide appeared to be necessary to improve the lifespan which would tend to indicate the ROS could be signalling molecules instead of molecular actors in ageing (Yang and Hekimi 2010). Similarly, work on Daf-2 mutants that are defective in insulin/IGF1-signalling, lead to the hypothesis that lifespan extension requires a transient increase in ROS. These act as signalling molecules to increase endogenous ROS defences and increased lifespan extending stress resistance (Zarse et al. 2012). Indeed, constitutive Daf-2 mutants have an increased resistance to paraquat treatment, an increased mitochondria metabolism and an increased activity of both SOD and catalase resulting in an overall decrease in ROS level. This signalling pathway involves the orthologues of AMPK, p38MAPK, NRF-2, PMK-1 and SKN-1.

In *C****aenorhabditis****.elegans* mildly inhibiting the mitochondrial respiratory chain, either genetically (Rea et al. 2007) or biochemically (De Haes et al. 2014) increases the lifespan of the worm, while more severe dysfunction shortens it (Rea et al. 2007). In tissue culture, inducing mitochondrial dysfunction may lead to cell cycle arrest and senescence, associated with an increase in mtDNA copy number (Wiley et al. 2016; Zelenka et al. 2015). However, intermittent treatment with 5 mM l-lactate (leading to ROS production, phosphorylation of AMPK and activation of PGC1-α) seems to improve the phenotype (Zelenka et al. 2015). The mitohormesis hypothesis (Ristow and Zarse 2010; Tapia 2006) can explain these disparate observations: a dysfunction coupled with a moderate increase in ROS activates protective quality control pathways and result in an overall improved mitochondria quality. However, continuous or strong MRC dysfunction leads to a substantial accumulation of ROS, which overwhelms the protective mechanisms. Similar ideas have been linked to the beneficial effect of mild stresses such as exercise and fasting (Zelenka et al. 2015; Tapia 2006).

## Mitochondria–nucleus relationship in ageing

### Mitochondria–nucleus crosstalk

Three decades ago the first pointers towards crosstalk between mitochondria and nucleus became evident, the abundance of nuclear-encoded transcripts appeared to vary with the mtDNA content (in quantity and quality) in budding yeast (Parikh et al. 1987). The concept of the retrograde response emerged, in which mitochondria signal dysfunction, thus inducing expression of nuclear genes. One such gene is CIT2 a peroxisomal isoform of citrate synthase, another is Rtg2p that activates the transcription factor Rtg1p–Rtg3p (Jia et al. 1997; Sekito et al. 2000). This retrograde response is triggered by a decrease in the mitochondrial membrane potential (Miceli et al. 2011) and is proportional to the extent of the mitochondrial dysfunction (Jazwinski 2005). While the precise details of the interaction between mitochondria and nucleus differ, there is ample evidence for such crosstalk in humans. For instance, many defects in mtDNA maintenance are caused by mutations in nuclear genes of the replisome (Naviaux et al. 1999; (Kaukonen et al. 2000; Spelbrink et al. 2001), and the mitochondrial dysfunction that results from mtDNA depletion or damage activates responses in a large number of nuclear genes (Hansson et al. 2004). Moreover, the interaction between mitochondria and nuclear DNA is important for longevity and ageing in humans because a non-random association between mtDNA and nuclear variability has been shown in centenarians (De Benedictis et al. 2000).

Much recent research has been carried out to investigate the role of epigenetics, and particularly of methylation, in the ageing process. Changes in the methylation pattern occur over the course of lifetime (Wang et al. 2012; Johansson et al. 2013; McClay et al. 2014). These changes can be either towards hypermethylation or hypomethylation (Johansson et al. 2013) with the CpG-rich promoters, preferential sites for methylation, tending to get hypermethylated and regulatory regions, poor in CpG, getting hypomethylated (Heyn et al. 2012). A study by Horvarth et al. has proposed the existence of an epigenetic clock formed by 343 CpG sites. Alterations at these sites are linked to increased risk of cancer and ageing phenotypes (Horvath 2013). The methylation status is the result of the effect of different factors such as sex, genetics, smoking and environmental constraints (Monick et al. 2012; Shenker et al. 2013; Shah et al. 2014).

Many studies have shown the importance of mitochondrial–nuclear crosstalk. In cultured cells, mitochondrial content is central to nuclear gene expression (Guantes et al. 2015; Muir et al. 2016); DNA methylation is linked not only to mtDNA copy number (Smiraglia et al. 2008) but also to the mtDNA haplotype (Bellizzi et al. 2012; Kelly et al. 2013). In addition, as most mitochondrial proteins are nuclear encoded, they can also be regulated at the epigenetic level; PolgA, for example, a nuclear-encoded mitochondrial protein involved in mtDNA replication, is dynamically regulated throughout development with its methylation status being negatively correlated with the mtDNA copy number (Kelly et al. 2012). It is thus likely that DNA epigenetics and mitochondrial function are controlling each other via a feedback loop (Koczor et al. 2013). Other DNA modifications, such as acetylation, also seem to be regulated by mitochondria and ATP levels (Wellen et al. 2009).

## The sirtuins play a key role in many of the processes that underlie ageing

Sirtuins, also known as Silent information regulator two (Sir2) proteins, regulate important biological pathways in eukaryotic cells and hence play a key role in many of the processes that underlie ageing. They have a histone deacetylase and mono-ribosyltransferase activity which is connected to the energy of the cell by sensing NAD:NADH ratio, NAD, NADH or nicotinamide levels. They affect modifications of histones and other proteins to regulate multiple cellular functions. Interest in their role in the regulation of lifespan and the ageing process began in 2001 with the finding that Sir2, encoding the sirtuin 1 protein (SIRT1), acts as a lifespan regulator in yeast. Sir2 overexpression increases lifespan (Tissenbaum and Guarente 2001) with mutations causing a severe reduction in lifespan (Kaeberlein et al. 1999). This result has been reproduced in mice and drosophila (Kanfi et al. 2012; Whitaker et al. 2013). Whether or not SIRT1 mediates the beneficial effects of caloric restriction on lifespan is still under debate (Kaeberlein et al. 2004; Tsuchiya et al. 2006). In humans, SIRT1 is involved in ageing and a positive correlation between its levels of expression and the mitotic activity has been established (Bai et al. 2014; Sasaki et al. 2006). Similarly its activation in mice does improve the lifespan of the animal (Mitchell et al. 2014).

The sirtuin family is composed of seven sirtuins with three localised to mitochondria including sirtuin 3 (SIRT3), which is localised to the mitochondrial matrix and has been implicated in age-related diseases (Zeng et al. 2014; Tao et al. 2014). This protein is involved in the regulation of mitochondrial ROS detoxification, ATP production and network dynamics by activation of the fusion protein OPA1, fatty acyl oxidation, metabolism and mitochondrial UPR (Jacobs et al. 2008; Ahn et al. 2008; Samant et al. 2014; Hirschey et al. 2010; Jing et al. 2013; Papa and Germain 2014). SIRT3 appears to be a mediator of caloric restriction as its expression and effect on mitochondrial protein acetylation is increased by 24 h of fasting (Jing et al. 2013). However, the exact role of SIRT3 in the ageing process remains unclear. Down-regulation of SIRT3 results in several age-related diseases including cancer, diabetes, cardiac pathologies and neurodegenerative disorders. In a mouse model, knocked out for SIRT3 (SIRT3 KO) an accelerated ageing phenotype is observed, mitochondrial integrity is lost, and MEFs are more prone to immortalisation (Kim et al. 2010). In this mouse model, a proportion of SIRT3 KO mice develop spontaneous cancer at old age. In humans, SIRT3 is found downregulated in several cancers, such as ovarian, breast, liver and gastric cancers (Finley et al. 2011; Zhang and Zhou 2012; Yang et al. 2014), potentially via an increased mitochondrial oxidative stress (Schieber and Chandel 2014; Kim et al. 2010) and associated changes in cell metabolism (Haigis et al. 2012).

As mentioned earlier, SIRT3 is also involved in metabolic disease such as diabetes. SIRT3 KO mice show an accumulation of lipids in liver and impaired fatty acid oxidation (Hirschey et al. 2010). They also develop a peripheral insulin resistance with a decreased PDH activity (Jing et al. 2013). Furthermore SIRT3 is implicated in preventing the metabolic shift away from fatty acid oxidation and towards glycolysis and in limiting the ROS production and the activity of transcription, and translation initiation factors involved in cardiac hypertrophy in the failing heart (Sundaresan et al. 2009).

Finally SIRT3’s role in mitochondrial function and high expression levels in the brain (Zeng et al. 2014; Ban et al. 2013; Sidorova-Darmos et al. 2014) make it a potential target for therapeutics aimed at preventing neurodegenerative disease. SIRT3 overexpression has been shown to protect neurons from cellular stresses such as ROS (Weir et al. 2012; Dai et al. 2014) and mitochondrial dysfunction and apoptosis due to SOD1 mutations (Song et al. 2013). SIRT3 reduction of ROS-associated damage is manifestly beneficial in mice as it slows down age-related hearing loss during caloric restriction (Someya et al. 2010).

### Caloric restriction and sirtuins

One strategy to limit the ageing effect is a calorie-restricted diet. This was first found to increase lifespan in mice (Sohal et al. 1994). The same study showed that age-related oxidative damage could be prevented by caloric restriction. These results were then confirmed in muscle from mice and heart from rats, where caloric restriction limits mitochondrial-associated oxidative damage (Lass et al. 1998; Gredilla et al. 2001). In yeast, caloric restriction extends lifespan by decreasing the NADH levels leading to an activation of Sir2 and a shift of the metabolism towards respiration (Lin et al. 2002, 2004). Overall caloric restriction seems to improve mitochondrial function, by enhancing the transcription of ROS scavengers and proteins linked to energy metabolism (Sreekumar et al. 2002). It protects mitochondria against mitochondrial DNA deletions (Cassano et al. 2004), against apoptosis by inducing SIRT1 (Cohen et al. 2004) and induces mitochondrial biogenesis (Lopez-Lluch et al. 2006; Civitarese et al. 2007) to improve mitochondrial function. Hypothetically since biogenesis is increased, degradation of dysfunctional mitochondria might also be increased; the first clue supporting this was that the expected decline in autophagy with age could be rescued by caloric restriction (Bergamini et al. 2003). More interestingly, a recent study has shown a protective effect of mitophagy on oxidative damage (Cui et al. 2013). Kidneys from rats under a calorie restriction diet displayed an increased mitophagy and less mitochondrial damage together with a decrease in a marker of senescence.

## Mitochondrial dynamics, quality control and ageing

### Mitochondrial fusion and fission and determinants of lifespan

Studies in yeast have shown that mitochondrial dynamics are an important determinant of lifespan (Bernhardt et al. 2015). This appears to depend on mitochondrial dynamics rather than the organisation of the mitochondrial network in itself (Bernhardt et al. 2015; Chen et al. 2005). In yeast, double mutants yeast for Mgm1 and Dnm1 have a mitochondrial network morphology similar to that of wild-type, their mitochondrial function is affected and their replicative lifespan is decreased (Bernhardt et al. 2015). Several other studies have linked fragmentation of the mitochondrial network with reduced lifespan (Braun and Westermann 2011; Aerts et al. 2008; Aerts et al. 2009). On the other hand, caloric restriction has been shown to decrease the expression of mitochondrial fission factors Drp1 and Fis1 and to upregulate the fusion factor Mgm1, resulting in a more filamentous network (Goldberg et al. 2009). In Rat, caloric restriction leads to the same effect and an increase in cristae number has been observed (Khraiwesh et al. 2014). These results are recapitulated in mammalian systems. For instance, a study of retina of young and old mice shows a change of mitochondrial dynamics in the retina with age: there is a decline of FIS1 and OPA1 in the ganglional cells and the outer plexiform layers. This contrasts with an increase in both OPA1 and FIS1 in the inner segment where damage is most likely to accumulate, suggesting a compensatory increase in mitochondrial turnover in this key region (Kam and Jeffery 2015). These results were further confirmed using electron microscopy revealing fragmented mitochondria in the retina. In a seminal study, Chen et al. showed that mitochondrial fusion is essential to maintain mitochondrial function in tissue culture. Loss of the mitochondrial fusion proteins OPA1 or MFN1 and 2 led to mitochondrial fragmentation, poor cell growth and impaired mitochondrial respiration (Chen et al. 2005). Mitochondrial fragmentation that did not significantly affect dynamics was insufficient to do this. Similarly, disrupting mitochondrial fission did not affect mitochondrial function or cell growth. However, in another study, inhibiting fission disrupted mitochondria segregation and was responsible for loss of properties (Katajisto et al. 2015). These investigators observed that in stem-like cells division is asymmetric; some cells inherit mainly “old” mitochondria and loose their stemness properties (self-renewal and pluripotency), whereas others get young mitochondria and stay stem-like. This indicates that a mechanism exists to sort old and young mitochondria and highlights the importance of mitochondrial turnover and quality in maintaining “stemness”.

Given that the cell uses several ways to maintain mitochondria (Rugarli and Langer 2012), what aspect of mitochondrial dynamics determine their propensity to age? MtDNA mutations tend to accumulate when the fusion/fission cycles are less frequent and there is less mtDNA mixing. Fusion appears to be important for mixing content of different mitochondrions (Tam et al. 2013; Chan 2012) hence keeping the network relatively homogeneous. It needs, however, to be opposed to enough counteracting fission events as there are numerous studies reporting that a fission arrest or a huge increase in fusion are detrimental. These include studies that show mitochondria hyperfilamentation that is apparent in brain from patients or mouse model of AD and leads to cellular senescence (Zhang et al. 2016; Park et al. 2010).

As well as affecting the fusion and fission of membranes, changes in mitochondrial dynamics also drive mitochondrial turnover. Hence mitochondrial hyperfusion can slow down axonal transport in neurons (Takihara et al. 2015) or inhibit mitophagy and worsen the quality of the pool of mitochondria (Park et al. 2010), which can lead to sarcopenia in muscle (Leduc-Gaudet et al. 2015). Increasing the expression of mitophagy protein PARKIN appears to improve mitochondrial turnover and reduce ageing in flies (Rana et al. 2013). Ferree et al. have shown that the turnover is also important to keep mitochondria “young” (Ferree et al. 2013). Recently resveratrol, which has a positive effect on lifespan, was shown to activate the Pink1/Parkin pathway by upregulating the glutathione levels in cells (Das et al. 2014). Finally, it is to be noted that lifelong caloric restriction results in a change in mitochondrial size and mass as well as ultrastructure (Khraiwesh et al. 2014). This also could be explained by a higher turnover rate and an overall improved mitochondrial quality.

### Effects of mitochondrial stress on lifespan can be rescued by UPRmt activation

UPRmt is a stress response triggered by the accumulation of unfolded protein in mitochondria. It promotes expression of mitochondrial chaperones (Durieux et al. 2011), limits protein import (Wrobel et al. 2015) and reduces mitochondrial translation (Haynes et al. 2013). The accumulation of unfolded proteins in mitochondria has been shown to induce nuclear genes encoding mitochondrial stress proteins such as Cpn60 and Cpn10 (Zhao et al. 2002). This response was shown to be transient and to correlate with the level of aggregates and is regulated by CHOP and C/EPBb. Some factors have been involved in UPRmt, such as HAF-1, a matrix protein exporter to export unfolded peptides (Haynes et al. 2010). Another mitochondrial stress sensor is the transcription factor, ATFS-1; in control conditions, it is imported in the mitochondria and degraded. When mitochondria are stressed, the import is less efficient and a fraction of ATFS-1 accumulates in the cytosol, allowing it to be imported in the nucleus, where it triggers transcription of the UPRmt genes (Nargund et al. 2012). It has been recently shown to bind to OXPHOS genes promoter. This limits the amount of transcripts during UPRmt and promotes the OXPHOS complex assembly and function (Nargund et al. 2015). Recently, CLK-1, a monooxygenase known to be mitochondrial and having a role in respiration and longevity, has been shown to translocate to the nucleus. This nuclear form of CLK-1 mediates a retrograde signalling pathway that responds to mitochondrial ROS. It regulates both mitochondrial ROS and UPRmt, and this signalling is conserved from worms to humans (Monaghan et al. 2015). The unfolded protein degradation into peptides involves the proteases CLPxP (Haynes et al. 2010) and Lon (Bezawork-Geleta et al. 2015). Lon appears to clear misfolded and not aggregated proteins (Bezawork-Geleta et al. 2015).

This stress response has been related to ageing and lifespan as, as stated earlier, a knockdown of CCO-1 in *C*.*elegans* moderately affects the ETC and increases longevity in an UPRmt-dependent way (Durieux et al. 2011). Work in fungi has also shed light on the involvement of UPRmt in longevity as an overexpression of the Lon protease in Podospora anserine leads to an increase in ATP-dependent serine protease activity. These fungi have less carbonaylated and carboxymethylated proteins and less H_2_O_2_ secreted resulting in an increase in lifespan and healthspan (Luce and Osiewacz 2009). Similarly mice knocked out for Surf1 (Surfeit locus protein 1, helps in the initial assembly of 13 subunits of the cytochrome c oxidase) are viable, displaying a decrease of more than 50 % in COX activity and have a longer lifespan. They appeared to have the same amount of ROS, membrane potential, ATP production and respiration in heart and muscle than controls. However, these mice have a mitochondrial energy metabolism decrease, which is combined to an increase in mitochondrial biogenesis markers, Nrf2 pathway and UPRmt. This indicates that mitochondrial dysfunction can lead to the induction of the mitochondrial stress pathway to confer protective effects (Pulliam et al. 2014). However, some work in *C*.*elegans* has recently challenged this relation between UPRmt and lifespan. There was no correlation between lifespan (positively or negatively) and UPRmt in an RNAi screen. Neither knockdown nor stabilisation of ATFS-1 affected the lifespan of the worms (Bennett et al. 2014).

### Mitophagy declines during ageing: from humans through to fungi

There is accumulating evidence that genetically increasing autophagy delays ageing in flies (Ulgherait et al. 2014), worms (Schiavi et al. 2015) and mice (Wu et al. 2013). Given that mitochondria are implicated in ageing, the increase in mitophagy that accompanies activation of autophagy could underlie this. Indeed, the importance of mitophagy in lifespan has been confirmed in drosophila where overexpression of Parkin leads to increased lifespan and decreased protein aggregation (Rana et al. 2013). Flies have decreased dMFN levels, fragmented mitochondria and show increased mitochondrial function. Parkin overexpression also extends neuron longevity and reduces protein aggregation in the brain. In *C.elegans*, mitophagy has been shown to be required for longevity under conditions of low-insulin/IGF-1 signalling or impaired mitochondrial function (Palikaras et al. 2015). Similarly feeding worms with lithium, an inducer of macroautophagy, improved lifespan and mitochondrial function (Tam et al. 2014). This group produced a mathematical model suggesting that lifespan can be extended by a combination of rapid mitochondrial fission, fusion and mitophagy to maintain mitochondrial function. This is also supported by the finding that mitophagy can be induced in response to frataxin depletion and that this increases lifespan of the worm (Schiavi et al. 2015).

The same kind of work has been carried out in the mouse where old mice have been fed with trehalose, a mitophagy inducer. These mice displayed an improvement of the levels of the mitochondrial quality controls mediators, especially PARKIN, BNIP3, SIRT3 and PGC1α, when compared to controls mice (LaRocca et al. 2014). Their artery walls do not stiffen and these results were reproduced ex vivo on arterial rings. As reported earlier, caloric restriction diet leads to increased mitophagy and decrease in both mitochondrial damage and markers of senescence in rats (Cui et al. 2013). The PolG mutator mouse, which accumulates mtDNA mutations and undergoes premature ageing and sarcopenia, displays evidence of increased autophagy with an increased expression of PGC1-α and mitochondrial fragmentation when compared to age-matched control (Joseph et al. 2013). In contrast to the other models discussed above, here the increased autophagy is insufficient to enhance mitochondrial quality in the presence of massive mtDNA damage (Li-Harms et al. 2015). Interestingly in humans, exercise, which has been shown to improve ageing phenotype, induces an increase in levels of protein involved in mitochondria biogenesis and mitochondrial dynamics (Konopka et al. 2014).

Hence, decreased or dysregulated mitophagy likely contributes to the decline in mitochondrial quality and function that leads to the ageing phenotype. A recent study has shown that in mouse and human, mitophagy is impaired during ageing in muscle satellite cells (Garcia-Prat et al. 2016). These are muscle-specific stem cells, hence characterised by self-renewal and long life span, potentially requiring mitophagy more than other cell types (Phadwal et al. 2013). This decrease of mitophagy could be due to an inflammation process as IL-10 null mice display a better ageing phenotype than the control weight mice (Ko et al. 2016).

Interestingly when mitophagy is impaired, mitochondria send a retrograde signal through SKN-1, a transcription factor that regulates both mitophagy and mitobiogenesis (Palikaras et al. 2015). This mitochondria–nuclear crosstalk important for mitochondria health and involving mitophagy has also been highlighted in a nucleotide excision DNA repair disorder leading to neurodegeneration (xeroderma pigmentosum group A). This disorder is characterised by impaired mitophagy due to excessive PINK1 cleavage (Fang et al. 2014). This excessive processing appears to be due to both an activation of PARP1 and an attenuation of the NAD(+)-SIRT1-PGC1α pathway.

As one would expect, coordination between biogenesis and recycling is needed to keep the pool of mitochondria healthy. The decline in mitophagy with ageing (Diot et al. 2015) thus disadvantages both the turnover of dysfunctional mitochondria and the production of fresh mitochondria, leading to a decreased life- and healthspan.

Finally, it is important to state that UPRmt and mitophagy are not working independently. Notably when unfolded proteins were expressed in the mitochondrial matrix, PINK1 accumulated on mitochondria able to maintain their membrane potential (Jin and Youle 2013). This resulted in a Parkin-dependant mitophagy. CLPxP is not needed for this response but a knockdown of LonP protease increases the accumulation of PINK1, which is not due to a decrease in membrane potential. In drosophila, a mild muscle mitochondrial distress preserved the mitochondria function as well as the muscle function and architecture and resulted in a prolonged lifespan (Owusu-Ansah et al. 2013). This beneficial result was abolished by an increase in anti-oxidant. Two pathways were identified; one through the UPRmt and the other one due to insulin signalling antagonisation which facilitates mitophagy (Owusu-Ansah et al. 2013). Forced expression of UPRmt genes, such as Hsp60, or overexpression of ImpL2 alone was sufficient to increase lifespan. The latter induced an increase in lysosome biogenesis and an increase in mitophagy was observed, manifest as mitochondria engulfed by autophagosomes.

## Conclusions

**Fig. 1:**
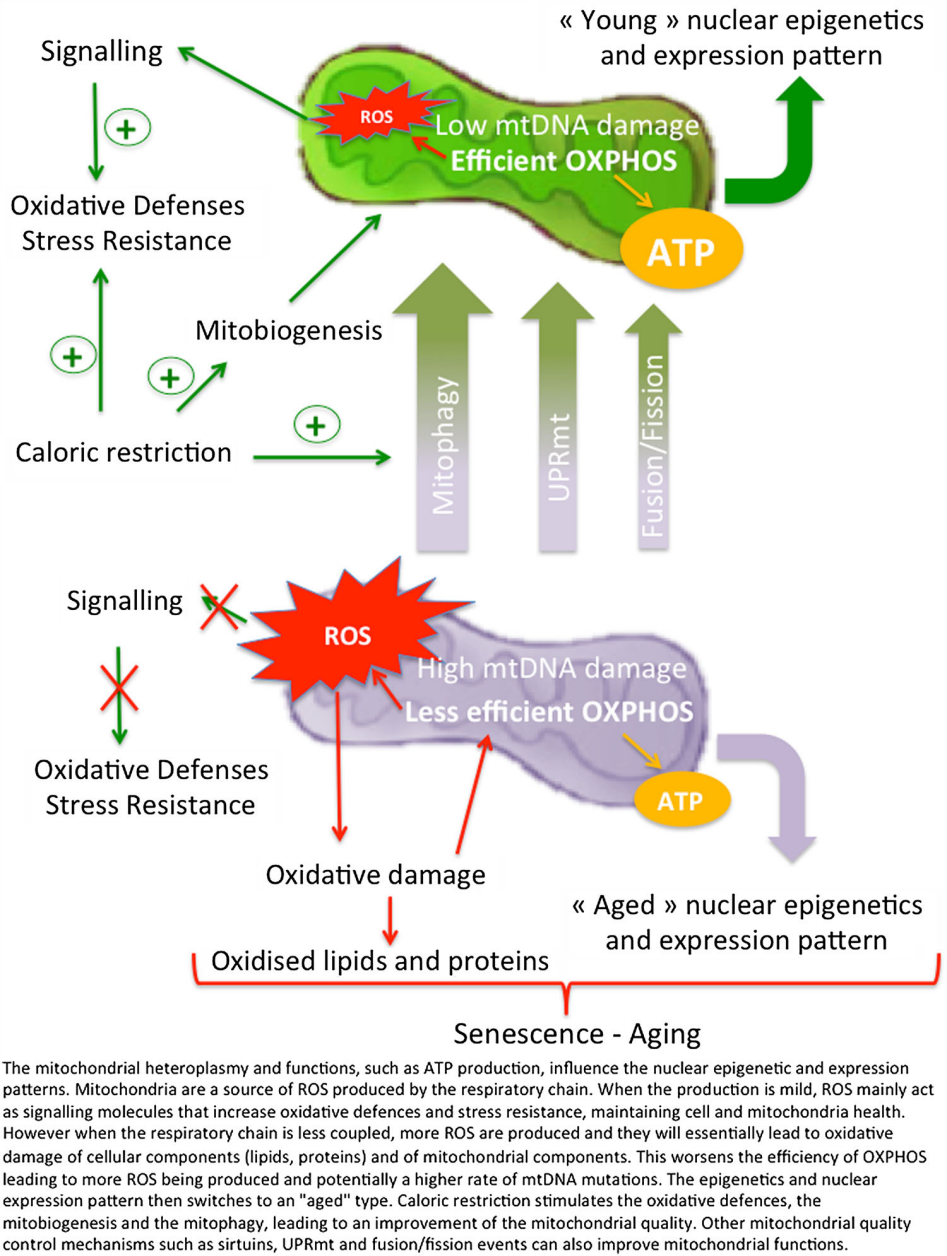
Mitochondria quality control improves the ageing phenotype

In this review, we have seen that mitochondria play a role in ageing at different levels (Fig. 1). First, the MRC as an important source of ROS that increases as mitochondrial quality declines. ROS production is indeed an important feature of the ageing process, whether it induces oxidative damage to proteins, lipids and DNA or acts as a signalling molecule.

Second, through their relationship with the nucleus, mitochondria affect nuclear gene expression. We now know that nuclear–mitochondria crosstalk is not only in the nucleus-to-mitochondria direction, via production and import of the vast majority of the proteins necessary to build a mitochondrion and regulation by sirtuins. The retrograde response where mitochondria content and activity regulate nuclear gene expression is also critically important (Guantes et al. 2015, 2016). Overall the mitochondria content and quality appear to be important features of the ageing process. This is highlighted by the importance of heteroplasmy for damaged mtDNAs, presumably due to a decreased efficiency in energy production leading not only to more ROS being produced but also to an effect on telomere as it has recently been suggested.

These observations convince us that mitochondria quality control has a very (maybe the most) important part to play in the ageing process. By modulating mitophagy, it may be possible to improve mitochondrial quality, limit mtDNA damage, regulate ROS production to what is necessary for signalling and keep the nuclear gene expression to the pattern and levels of healthy young cells.

UPRmt is one of these quality control pathways although its role in longevity has been recently challenged. Stimulating mitochondrial dynamics to keep a homogenous functional mitochondrial network is another strategy that might prolong the “young” state of cells. Mitophagy coupled to mitobiogenesis stands out, however, as the most promising, unambiguous and potent way to keep unaltered mitochondria and thus “young” cells. Learning how to stimulate mitophagy/mitobiogenesis should represent an important research axis in therapeutics towards ageing-related disease.
